# Ornamental Plants and Urban Gardening II

**DOI:** 10.3390/plants14233653

**Published:** 2025-11-30

**Authors:** Szilvia Kisvarga, László Orlóci

**Affiliations:** Institute of Landscape Architecture, Urban Planning and Garden Art, Hungarian University of Agriculture and Life Sciences (MATE), 1118 Budapest, Hungary

## 1. Genetic and Physiological Developments in Ornamental Plant Breeding

One flagship of the ornamental plant industry is the orchid; it is no coincidence that a comprehensive review focuses on the breeding, cultivation, and potential applications of *Phalaenopsis.* Han et al. [1] clearly show how classical hybridization has developed into the 21st century: mutation breeding techniques and tissue culture systems today not only support the creation of special flower colors and forms but also target selection for climatic tolerance. The authors connect industry trends with concrete points of breeding practice: compact habits, long-lasting flowering, stable performance under changing indoor and semi-protected outdoor conditions are now just as much “selection goals” as pigmentation or fragrance. From an editorial point of view, this change in approach is essential: the story of *Phalaenopsis* is not about “luxury decoration” but about how an ornamental plant becomes a functional urban element, serving both comfort and sustainability needs. A related study is presented by Kovács et al. [2], who optimized in vitro regeneration of *Adenophora liliifolia* using combined plant-growth-regulator treatments, natural biostimulant extracts, and fine-tuned medium pH. Although developed for a protected wild species, their protocol exemplifies how precise control of endogenous hormonal balance can enhance regeneration efficiency and stress resilience—a principle directly transferable to ornamental crop improvement. By coupling physiological stability with efficient propagation, this work combines ex situ conservation, commercial production, and climate-oriented breeding goals.

The rose remains a “cult plant” in European and global urban green spaces, and several papers in this issue shed new light on it. Crossing experiments investigate the inheritance of traits between hybrid tea roses and old garden roses [3]. The practical significance is clear: predicting how agronomic and ornamental traits (such as flower form, fragrance, and growth vigor) combine can directly shape the urban plant palettes. Mapping this “mosaic” of inheritance helps reduce blind crosses and brings breeders closer to the goal of durable, ornamental, and increasingly stress-tolerant cultivars for public plantings.

To capture aesthetic quality objectively and measurably, a measurement-based petal color system was developed for cultivated roses. This “colorimetrically balanced” approach creates quantitative categories that are directly usable in horticultural practice: color fidelity, selection between color classes, and consistent communication all help ensure that public procurement and maintenance decisions rely on measurable parameters rather than subjective descriptions. Thus, Boronkay et al. [4] provide more than just a “color system”: they aim to equip sustainable urban green infrastructure with a quality assurance tool that supports both biodiversity and visual coherence.

A critical genetic–molecular factor under climate change is understanding the regulatory networks behind heat and other abiotic stresses. In *Rosa* hybrids, a heat stress transcription factor was identified and functionally characterized. The nucleus-localized, transcriptionally active protein shows that silencing leads to heat sensitivity, while transient overexpression increases heat tolerance. This is significant in two ways. First, it provides a concrete molecular target for breeding, with direct measurable impact on urban heat conditions (flower quality, leaf condition, and sustained ornamental value during heat waves). Second, it sets a methodological benchmark: adding functional genomics to phenotypic selection, which has long been dominant in ornamentals—a toolkit previously typical of major crop species [5]. Regarding methodological expansion, Kisvarga et al. [6] investigated more than two-hundred-year-old *Ginkgo biloba* specimens exposed to contrasting urban microclimates. Through combined micromorphological, enzymatic, and miRNA analyses, the study revealed adaptive traits such as altered stomatal density, enhanced peroxidase activity, and stress-response small-RNA expression patterns. These findings confirm that ancient trees embody long-term genetic and epigenetic adjustments to urban stressors, positioning *Ginkgo biloba* as a benchmark species for understanding and utilizing tree longevity and plasticity in city environments.

Among ornamental trees, magnolias deserve special attention; Zhou et al. [7] analyzed a yellow–green leaf mutant of *Magnolia* with physiological, cytological, and transcriptomic depth. Reduced chlorophyll-b, a shifted chlorophyll a/b ratio, and disturbed thylakoid architecture together explain both altered leaf coloration and photosynthetic performance; thousands of differentially expressed genes highlight pathways (pigment biosynthesis, photosynthesis, protective mechanisms) where the ornamental phenotype’s physiological cost and benefit develop hand in hand. The message is clear: unusual foliage color is not a goal in itself but a complex trait package that must be interpreted in light of urban applicability. From an editorial perspective, this is the level of understanding that allows “specialness” to strengthen rather than weaken urban performance.

The success of breeding ultimately depends on the physiological and nutritional environment in which planting and early maintenance take place. While detailed stress-physiology materials are discussed in the next section, here—to close the genetic perspective—we highlight an instructive study [8] on *Podocarpus macrophyllus*. The researchers show how light quality (including urban street lighting and night-time artificial illumination) and exponential nutrient pre-feeding influence internal nitrogen redistribution, chlorophyll content, and the post-planting vitality of trees. In the urban context, where street lighting is unavoidable, this knowledge can be turned into fertilization and light-management protocols: less dieback, fewer replacements, and more stable early growth.

## 2. From Physiology to Urban Performance: Environmental Constraints and Soil Biology

The genetic development of ornamentals only becomes a true urban success story if we understand the environmental challenges they face. The city is not a sterile lab: polluted, compacted, nutrient-poor, and salt-loaded soils, extreme temperature fluctuations, water scarcity, and variable light conditions shape plant life. One of the greatest strengths of this Special Issue is that several papers reveal these factors with deep physiological and soil-biological investigations. These works remind us that sustainable urban greenery depends not only on plant genetics but also on the invisible ecological network of roots, microorganisms, and soil processes that sustain long-term performance. Moreno-García et al. [9] extended this principle by developing a high-throughput micropropagation system for *Sedum sediforme* and *S. album*, two drought-tolerant Mediterranean succulents with proven suitability for extensive green-roof installations. Their optimized in vitro protocol achieved shoot proliferation rates above 60% and produced physiologically uniform plantlets capable of rapid acclimatization under low-substrate and high-temperature stress. This research demonstrates that ornamental propagation and ecological functionality can converge with clonal propagation of robust genotypes, ensuring predictable rooftop performance, improved water-use efficiency, and reduced maintenance cost, which are all key parameters of urban greening resilience.

A key point here is the role of mycorrhizal fungi and the soil biota. Papp et al. [10] highlight the ecological functions of arbuscular mycorrhizal fungi (AMF), which can also be used during urban soil restoration. Their symbiotic relationship with roots enhances water and nutrient uptake and increases drought tolerance and heavy metal resistance. Field and experimental results show how AMF inoculation can improve the initial success of plantings on degraded urban soils. From an editorial perspective, this is paradigm-shifting: in cities, it is not enough to “choose a good cultivar”. Without ecological soil rehabilitation, even the best genetics may fail. This insight can reshape urban green infrastructure protocols, making AMF treatment as basic as improvements in physical soil.

Light quality and nutrient strategy are further detailed in the *Podocarpus macrophyllus* pre-cultivation study mentioned above. Urban light pollution and night-time LED lighting are new, long-overlooked stress factors. Wang et al. [8] show that both the wavelength and intensity of light during nursery production fundamentally affect nitrogen redistribution, chlorophyll levels, and post-planting vitality. Exponential nitrogen feeding—rather than a single large fertilizer dose—increases survival and early growth. Such knowledge can be directly integrated into urban tree planting practice, leading to fewer planting failures, with both economic and ecological benefits.

Soil contamination is another key challenge: Di et al. [11] investigated the response of *Rhododendron simsii* to cadmium stress when treated with melatonin. Using JIP tests, chlorophyll fluorescence, and pigment analysis, the authors show that melatonin significantly improves photosynthetic efficiency and reduces oxidative stress caused by cadmium. While at first glance this may seem like a niche issue, urban soils often face heavy metal pollution (industrial past, traffic-related deposition). The message is twofold: phytohormone-based treatments may help during critical establishment periods, and urban soil toxicity now demands not only soil improvement but also physiological support strategies.

Indoor and semi-intensive green spaces form another research direction with increasing importance in cities. Eisa et al. [12] examine *Vriesea splendens* under different substrate compositions. The results show that water retention capacity, nutrient profile, and root zone aeration decisively influence physiological performance and ornamental value. For green roofs, indoor living walls, or greenhouse installations, substrate parameters are critical: sustainability depends not only on species choice but also on the right substrate strategy. From an editorial viewpoint, this emphasizes that urban indoor and semi-protected greening is no longer just about design—it is a matter of precision cultivation technology.

Another often underestimated aspect of the urban climate is phenology and the behavior of fast-growing species. A detailed study by Chan et al. [13] on bamboo examines their growth and phenological rhythms. Rapidly spreading, high-biomass species are increasingly used in parks and protective green belts, but they carry invasion risk. Refined phenological models could help plan maintenance and predict ecological impact. From an editorial perspective, climate change forces us to rethink the role of fast-growing species: their dense cover and transpiration are valuable, but only if invasion risk and maintenance costs are well understood.

Halophytic plants also deserve attention: salinizing soils are becoming relevant even in urban contexts due to irrigation water quality, road de-icing salts, and industrial land reclamation. Detailed morphological and physiological studies of local salt-tolerant species show that utilizing native halophyte genetic resources can be a real option for greening extreme urban sites. This approach—local adaptation and ecologically native stress-tolerant plants—is both sustainable and biodiversity-friendly. Liang et al. [14] expand this perspective by evaluating *Rhododendron* species through a multi-criteria Analytic Hierarchy Process that integrates ornamental, ecological, and adaptive traits. Rather than treating aesthetic appeal as an isolated criterion, their framework quantifies resilience-related parameters—cold and drought tolerance, flowering stability, and maintenance demand—alongside color and form. This approach demonstrates how data-driven preference modeling can refine species selection for urban plantings, ensuring that ornamental beauty aligns with functional sustainability.

## 3. Diversity, Pollination, and Allergenicity in Urban Landscapes

Maintaining biodiversity in cities is one of the greatest challenges. Poje et al. [15] analyze how allergenic plants are represented in urban communication, especially through online visual content. By systematizing hundreds of plant photos available on the internet, it was found that most images depict species with low allergenic potential, while highly allergenic species rarely appear. This “invisibility” poses a public health risk: citizens do not learn to recognize problematic species and, thus, cannot avoid pollen exposure. From an editorial perspective, this is an important gap-filling study: without digital visual education, it is difficult to overcome societal barriers to allergen reduction. This work also offers practical recommendations in that municipal and professional communication should highlight highly allergenic species more explicitly—not to create fear, but to raise awareness.

In support of biodiversity and pollinators, another innovative study investigates nectar source diversity in urban green areas within a phylogenetic framework. The key finding is that while urban green spaces as a whole do not produce more nectar than agricultural or natural areas, their nectar diversity is significantly higher, mainly because of non-native ornamentals. This research poses a new question: is there a hidden, phylogeny-based “selection logic” behind the introduction of non-native plants to cities? Results suggest that the evolutionary patterns of nectar sugar variation may be more important than visible flower traits. This insight is valuable: it helps us understand why urban horticulture is drawn to certain exotic species and how pollinator-friendly design can become more intentional if functional and evolutionary patterns are considered. From an editorial point of view, this work points toward fine-tuning biodiversity planning: “bee-friendly” labels are not enough. Instead, we need to understand the evolutionary and nectar-provision strategies behind plant choices.

## 4. Digital and Design Innovations in Urban Green Infrastructure

Digital technology is also transforming landscape architecture. Yuan et al. [16] introduce VR-based design methods for green roofs in coastal cities, allowing designers, users, and decision-makers to “walk through” and evaluate spaces before construction. This reduces mistakes, improves user experience, and optimizes climate-adaptive design. The editorial message is clear: the future of green infrastructure design is interactive and data-driven.

Another innovative approach uses multimodal interaction and computer vision to develop planning frameworks for urban botanic gardens in coastal areas. Integrating visual and psychological user needs can elevate the design of green spaces to a new level, making them not just beautiful but experience-rich and functional landscapes. This is particularly important in tourism and recreation zones, where green spaces carry both ecological and economic value.

From an editorial standpoint, the greatest strength of this Special Issue is its integrative vision. The papers presented here move beyond isolated plant lists or single-discipline solutions. Instead, they create a system-level understanding: cities are not only planting trees but building living habitats.

Genetics and molecular physiology show us how to create plants that survive in the urban environment. Soil ecology and stress physiology teach us how to make those plants function and thrive under real, often harsh city conditions. Biodiversity-focused, socially aware, and technologically innovative approaches show why this work matters—how urban greenery can gain public acceptance, cultural meaning, and long-term support.

If these insights can be translated into practice, future urban green spaces will be more than decorative plantings. They will be resilient ecosystems that withstand climate stress, support biodiversity, remain economically maintainable, and connect deeply with the communities they serve.

## References

[1] Han C., Dong F., Qi Y., Wang Y., Zhu J., Li B., Zhang L., Lv X., Wang J. (2025). The Breeding, Cultivation, and Potential Applications of Ornamental Orchids with a Focus on Phalaenopsis—A Brief Review. Plants.

[2] Kovács Z., Portocarrero L.K., Honfi P., Kohut I., Eisa E.A., Tilly-Mándy A. (2024). Enhancing Micropropagation of Adenophora liliifolia: Insights from PGRs, Natural Extracts, and pH Optimization. Plants.

[3] Kılıç T., Kazaz S., Meral E.D., Kırbay E. (2024). Inheritance of Some Traits in Crosses between Hybrid Tea Roses and Old Garden Roses. Plants.

[4] Boronkay G., Hamar-Farkas D., Kisvarga S., Békefi Z., Neményi A., Orlóci L. (2024). Developing a Colorimetrically Balanced, Measurement-Based Petal Colour System for Cultivated Rose (*Rosa* L. Cultivars) and the Resulting Colour Categories. Plants.

[5] Sun Y., Li S., Wu X., Zhu J., Dong F., Pei Z., Li Z., Zhao S., Wang C. (2025). Characterization and Functional Analysis of RhHsfA7, a Heat Stress Transcription Factor in Roses (*Rosa hybrid* ‘Samantha’). Plants.

[6] Kisvarga S., Hamar-Farkas D., Horotán K., Gyuricza C., Ražná K., Kučka M., Harenčár Ľ., Neményi A., Lantos C., Pauk J. (2024). Investigation of a Perspective Urban Tree Species, Ginkgo biloba L.; by Scientific Analysis of Historical Old Specimens. Plants.

[7] Zhou X., Yang S., Zhou F., Xu L., Shi C., He Q. (2025). Physiological, Cytological and Transcriptome Analysis of a Yellow–Green Leaf Mutant in *Magnolia sinostellata*. Plants.

[8] Wang Y., Sun X., Li S. (2024). Light Quality Effect on Internal N Retranslocation in *Podocarpus macrophyllus* Precultured with Exponential Nutrient Loading. Plants.

[9] Moreno-García I., García-Sogo B., Soler S., Rodríguez-Burruezo A., Moreno V., Pineda B. (2025). Efficient Micropropagation of *Sedum sediforme* and *S. album* for Large-Scale Propagation and Integration into Green Roof Systems. Plants.

[10] Papp L., Habtemariam A.A., Brandt S., Cseh P., Heller Á., Péter B., Szakály Á.P., Kiszel P., Codogno B., Bratek Z. (2023). A Possible Perspective of Recultivation with Arbuscular Mycorrhiza-Inoculated Drought-Tolerant Herbaceous Plants. Plants.

[11] Di H., Liang Y., Gong Y., Jin S., Xu Y. (2025). The Effect of exogenous melatonin on the photosynthetic characteristics of *Rhododendron simsii* under cadmium stress. Plants.

[12] Eisa E.A., Pasquel Davila D.S., Ördögh M. (2025). Enhancing acclimatization conditions for *Vriesea splendens* ‘fire’: A comparative analysis of substrate effects on growth and survival. Plants.

[13] Chan K.N., Veres A., Liang Z., Kisvarga S., Neményi A. (2024). A Shoot Phenological Study of Certain Phyllostachys Bamboo Taxa Under Central European Climatic Conditions. Plants.

[14] Liang J., Chen Y., Tang X., Lu Y., Yu J., Wang Z., Zhang Z., Ji H., Li Y., Wu P. (2024). Comprehensive evaluation of appreciation of Rhododendron based on analytic hierarchy process. Plants.

[15] Poje M., Vukelić A., Židovec V., Prebeg T., Kušen M. (2024). Perception of the vegetation elements of urban green spaces with a focus on flower beds. Plants.

[16] Yuan J., Kim C.S. (2024). The ecological design of marine urban green space plant landscaping based on the concept of sustainability. Plants.

